# The role of digestive system diseases in cerebrovascular disease: a comprehensive Mendelian randomization study

**DOI:** 10.3389/fneur.2024.1389352

**Published:** 2024-05-24

**Authors:** Hao Qin, Shihuan Suo, Fan Yang, Pengfei Hao, Xianfeng Zhang

**Affiliations:** ^1^Department of Neurosurgery, The First Hospital of Jilin University, Changchun, China; ^2^Department of Anesthesiology, The First Hospital of Jilin University, Changchun, China; ^3^Department of Neurosurgery, Tongji Shanxi Hospital, Shanxi Bethune Hospital, Shanxi Academy of Medical Sciences, Third Hospital of Shanxi Medical University, Taiyuan, China

**Keywords:** digestive system diseases, stroke, intracranial aneurysm, Mendelian randomization, causal relationship

## Abstract

**Background:**

Cerebrovascular disease, among the most prevalent neurological disorders, poses a substantial threat to human health with its elevated mortality and disability rates, placing considerable strain on healthcare systems. Although several studies in recent years have suggested a potential association between digestive system diseases and cerebrovascular diseases, the findings remain inconsistent.

**Methods:**

Genome-wide association study (GWAS) summary data for 12 digestive diseases and cerebrovascular diseases were used to conduct Mendelian randomization (MR) analysis. In this investigation, we endeavored to elucidate the causal relationship between digestive system diseases and cerebrovascular diseases. Employing a comprehensive approach, including two-sample MR (TSMR), multivariate MR (MVMR), and two-step MR analysis, we leveraged summary statistics data obtained from published GWAS. The primary analysis method employed was inverse variance weighted (IVW), with MR-Egger and weighted median (WM) as secondary methods. Sensitivity analysis included heterogeneity testing, horizontal multivariate testing, MR-PRESSO, and a “leave-one-out” method. Additionally, the *F*-statistic was utilized to assess the strength of instrumental variables, ensuring robust results.

**Results:**

In the TSMR analysis, this study found a significant causal relationship between genetically predicted gastroesophageal reflux disease (GERD) and any stroke (AS), any ischemic stroke (AIS), large-artery atherosclerotic stroke (LAS), intracranial aneurysm (IA), and subarachnoid hemorrhage (SAH). In MVMR analysis, this study found that even after adjusting for systolic blood pressure (SBP), body mass index (BMI) and type 2 diabetes (T2D), the causal relationship remains exist. In the two-step MR mediation analysis, it was found that BMI, SBP and T2D play mediating role in the causal relationship between GERD and cerebrovascular diseases.

**Conclusion:**

This study indicates a clear positive causal relationship between GERD and cerebrovascular diseases, and this causal association remains significant even after adjusting for BMI, SBP and T2D. The mediation MR analysis suggests that BMI, SBP and T2D may mediate the causal relationship between GERD and the risk of cerebrovascular diseases.

## Introduction

Digestive and cerebrovascular diseases are two major disease categories affecting global health, and they involve, respectively, the body’s digestive and circulatory systems. The state of health of these two systems is closely linked, and a number of studies are gradually revealing the complex relationships that may exist between them.

Digestive disorders, such as inflammatory bowel disease (IBD), liver disease, and GERD, encompass a variety of conditions that impact food digestion, absorption, and excretion. This group of diseases is increasingly prevalent worldwide, garnering significant attention within the medical community. In contrast, cerebrovascular diseases, including strokes and cerebral aneurysms, result in disruptions or abnormalities in blood supply to the brain. While traditionally associated with elderly individuals, cerebrovascular diseases are now affecting younger and middle-aged populations more frequently, imposing a substantial burden on the healthcare system. These conditions are not only a leading cause of disability and mortality (1) but also contribute significantly to the overall social health system challenges (2).

Several studies have examined the association between various digestive disorders and the risk of stroke. In a prospective study involving Taiwanese individuals, the incidence of stroke, including ischemic and hemorrhagic strokes, was notably higher in those with gallstone disease compared to those without (3). A separate prospective study investigating the relationship between peptic ulcer and stroke found that peptic ulcer increased the risk of large atherosclerotic stroke, even after adjusting for factors such as age, gender, hypertension, and diabetes (4). Furthermore, research has indicated that patients diagnosed with Crohn’s disease (CD) may have a heightened risk of stroke based on several studies (5–7). A combined analysis of patients with CD and ulcerative colitis (UC) revealed that 1.3 to 6.4% of adults with inflammatory bowel disease (IBD) and 3.3% of children with IBD experienced cerebrovascular complications (8). Similarly, Ludvigsson’s et al. (9) study identified a positive correlation between celiac disease and cerebral hemorrhage and ischemic stroke. However, conflicting findings emerged from another study, which reported no significant disparity in stroke risk between adults with celiac disease and the general population (10). Despite the collective body of literature investigating the link between digestive disorders and cerebrovascular disease, the findings are often influenced by multiple confounding variables, thus complicating the ability to establish consistent outcomes.

In traditional observational epidemiologic studies, causal inference is often hindered by confounding and the potential for reverse causation (11). However, the method of Mendelian randomization (MR) offers an alternative approach to uncovering causal relationships between exposures and outcomes. Just as in the case of Mendel’s laws of inheritance, genes are passed on randomly from parents to offspring, leading to offspring genotypes that are typically uncorrelated with confounding variables in the population. Furthermore, genotypes are established at the moment of conception and remain constant, thus circumventing the issue of reverse causation. Previous observational studies have identified a correlation between certain digestive diseases and a heightened likelihood of developing cerebrovascular disease. Our study is to explore the causal relationship between digestive system diseases and cerebrovascular diseases through two-sample MR, multivariate MR, and mediated MR. By utilizing MR, this research methodology bypasses the impact of confounding variables often present in observational studies, leading to more reliable outcomes. Consequently, these findings offer valuable insights for clinical strategies aimed at mitigating the risk of cerebrovascular disease subsequent to digestive disorders.

## Methods

Detailed information of all GWAS data was listed in Table 1.

**Table 1:** GWAS summary data information.

Characteristic	Resource	Sample size	Population ancestry	PMID
GERD	IEU	602,604	European	34187846
GU	IEU	474,278	European	34594039
Gastric cancer	IEU	347,406	European	34594039
Celiac disease	IEU	23,649	European	22057235
CD	IEU	20,883	European	26192919
UC	IEU	417,932	European	34594039
Cirrhosis	IEU	347,406	European	34594039
NAFLD	GWAS catalog	778,614	European	34841290
Cholecystitis	GWAS catalog	456,348	European	34737426
PBC	IEU	24,510	European	34033851
AP	IEU	479,902	European	34594039
CP	IEU	477,528	European	34594039
SBP	IEU	385,798	European	34017140
BMI	IEU	461,460	European	——
T2D	IEU	655,666	European	30054458
AS	METASTROKE	446,696	European	29531354
AIS	METASTROKE	440,328	European	29531354
CES	METASTROKE	211,763	European	29531354
LAS	METASTROKE	150,765	European	29531354
SVS	METASTROKE	198,048	European	29531354
IA	——	317,636	European	33199917
uIA	——	317,636	European	33199917
SAH	——	317,636	European	33199917

GERD, gastroesophageal reflux disease; GC, gastric ulcer; CD, Crohn’s disease; UC, ulcerative colitis; NAFLD, non-alcoholic fatty liver disease; PBC, primary biliary cholangitis; AP, acute pancreatitis; CP, chronic pancreatitis; SBP, systolic blood pressure; BMI, body mass index; T2D, type 2 diabetes; AS, any stroke; AIS, any ischemic stroke; CES, cardioembolic stroke; LAS, large-artery atherosclerotic stroke; SVS, small-vessel stroke; IA, intracranial aneurysm; uIA, unruptured intracranial aneurysm; SAH, subarachnoid hemorrhage.

### Digestive system diseases data source

GWAS data for GERD, gastric ulcer (GU), gastric cancer, celiac disease, UC, CD, acute pancreatitis (AP), chronic pancreatitis (CP), cirrhosis of the liver, and primary biliary cholangitis (PBC) were obtained from the Integrated Epidemiology Unit open Genome-Wide Association Study (IEU open GWAS) database (website: https://gwas.mrcieu.ac.uk/) in the present study; for non-alcoholic fatty liver disease (NAFLD), from the GWAS catalog (website: https://www.ebi.ac.uk/gwas/home); and for cholecystitis, from the UK Biobank (website: https://pan.ukbb.broadinstitute.org/). The sample size ranged from 20,883-778,614. All studies are multi-centre, large-sample, repeatedly validated gene-disease association studies conducted at the genome-wide level.

### Stroke data sources

The GWAS summary data for stroke were sourced from the MEGASTROKE Consortium (12). This study comprised 29 investigations, encompassing a total of 67,162 cases and 454,450 controls. The MEGASTROKE Consortium defined stroke as a rapidly developing focal (or global) disturbance of cerebral function lasting more than 24 h or leading to death, with no apparent cause other than vascular origin. The MEGASTROKE Consortium classified strokes into AS (40,585 cases and 406,111 controls), AIS (34,217 cases and 406,111 controls), LAS (4,373 cases and 146,392 controls), CES (7,193 cases and 204,570 controls), and SVS (5,386 cases and 192,662 controls) according to the Trial of Org 10,172 in acute stroke treatment classification. To reduce population stratification bias, we restricted the stroke population to individuals of European ancestry.

### Intracranial aneurysm data sources

The summary statistics for individuals of European ancestry with intracranial aneurysms were obtained from a genome-wide association study (13). This study comprised 23 cohorts, totaling 79,429 individuals. It included three combined datasets: GWAS summary data for all intracranial aneurysms (ruptured, unruptured, and unknown rupture status) (*n* = 7,495); for unruptured intracranial aneurysms (*n* = 2,070), and for aneurysmal subarachnoid hemorrhage (aSAH) (*n* = 5,140).

### Metabolic factors data sources

The GWAS data for systolic blood pressure (SBP), body mass index (BMI) and type 2 diabetes (T2D) are sourced from the IEU GWAS database. The sample size for systolic blood pressure is 385,798, for BMI, it is 461,460, and for T2D, it is 655,666.

### Study design

**Figure 1:**
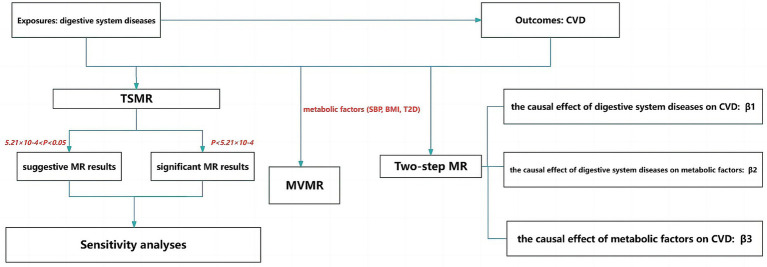
Flow chart of the study. TSMR, two-sample MR; MVMR, multivariate MR; CVD, cerebrovascular diseases.

As shown in Figure 1, firstly, this study used two-sample Mendelian randomization (TSMR) analysis to explore the causal relationship between digestive system diseases and cerebrovascular diseases. The Bonferroni correction method was applied for multiple testing corrections, with a significance threshold set at *p* < 5.21 × 10^−4^ considered a significant causal relationship, and 5.21 × 10^−4^ < *p* < 0.05 considered a suggestive causal relationship. Secondly, a significant exposure factors identified from the TSMR results and metabolic factors (SBP, BMI and T2D) were subjected to a MVMR analysis with cerebrovascular diseases. Thirdly, a two-step MR analysis was employed to explore whether metabolic factors play a mediating role between genetically predicted digestive system diseases and cerebrovascular diseases. Separate two-sample MR analyses were conducted to investigate the causal relationships between digestive system diseases and metabolic factors, as well as between metabolic factors and cerebrovascular diseases. To determine the mediating role of metabolic factors in the causal relationship between digestive system diseases and cerebrovascular diseases, the causal effect between genetically predicted digestive system diseases and cerebrovascular diseases, referred to as the total effect, was defined as β1. The causal effect of digestive system diseases on metabolic factors was defined as β2, and the causal effect of metabolic factors on cerebrovascular diseases was defined as β3. Here, β2 × β3 represents the mediation effect, and β2 × β3/β1 represents the percentage of the mediating effect.

### Instrumental variable selection

In order for genetic variation to qualify as a valid IV for causal inference in MR studies, three core assumptions must be satisfied. First, genetic variation must be strongly correlated with exposure. Secondly, genetic variation should not be associated with confounders of the exposure and outcome relationship. Thirdly, genetic variation should only be associated with outcomes through the exposure being studied. Therefore, we select single nucleotide polymorphisms (SNPs) associated with exposure factors at the genome-wide significance level (*p* < 5 × 10^−8^). And Linkage disequilibrium (LD) was removed with *R*^2^ < 0.001 and clumping window size >10,000 kb. SNPs with minor allele frequency (MAF) ≤0.01 and with palindrome were excluded. The *F*-statistic (beta^2^/se^2^) was used to assess the power of every SNP. SNPs with *F*-statistic <10 were excluded. Finally, we excluded SNPs that were associated with confounders or outcomes according to the PhenoScanner database.^1^

### Statistical analysis

We perform MR analysis using the TwoSampleMR package and MR-PRESSO package in R software (version 4.2.2).

In this study, many methods were employed to assess the causal relationship between digestive system diseases and cerebrovascular diseases, including the Wald ratio method, inverse variance weighted (IVW), MR-Egger method, and weighted median method (WM).

The Wald ratio method is a primary approach that uses a single SNP as IV to assess the causal relationship between exposure and outcome. Additionally, when more than one IV is available, this method can be used to calculate the causal effect for each IV. The IVW method is the main method in our study. When genetic variations are effective IVs, each variant provides a consistent estimate of the causal effect. Therefore, IVW method usually provides a consistent estimate of the causal effect of the risk factor on the outcome only when all genetic variations are effective IVs (14). If one or more variants are ineffective, the estimate may be biased (14). MR-Egger method and WM are used as secondary methods. The MR-Egger method not only provides consistent estimates of causal effects under InSIDE (INstrument Strength Independent of Direct Effect) assumption but also assesses the presence of horizontal pleiotropy. Under the InSIDE assumption, the intercept of the MR-Egger analysis can be interpreted as the average pleiotropic effect of the included genetic variations. If the average pleiotropic effect is zero, IVW method gives consistent estimates of the causal effect. Conversely, if the intercept of the MR-Egger analysis is non-zero, indicating a non-zero average pleiotropic effect, or if the InSIDE assumption is violated, it suggests bias in the IVW estimates. Additionally, testing the intercept of the MR-Egger analysis can evaluate the level of horizontal pleiotropy in the instrumental variables (15). Similarly, the weighted median method often yields robust estimates when up to 50% of SNPs are ineffective IVs (16).

### Sensitivity analysis

To ensure the impartial results, a series of sensitivity analyses were performed. Cochran’s IVW *Q* statistic was used to quantify the heterogeneity of the IVs. And *p*-value >0.05 of Cochran’s IVW *Q* indicated no heterogeneity. Additionally, the MR-Egger regression intercept method was employed to estimate horizontal pleiotropy of SNPs, with a *p*-value >0.05 suggesting no horizontal pleiotropy (17). In the presence of horizontal pleiotropy, MR-Pleiotropy Residual Sum and Outlier (MR-PRESSO) were used to further detect it. If the MR-PRESSO global test indicated the presence of horizontal pleiotropy, SNPs with outliers demonstrating horizontal pleiotropy were excluded, and the IVW analysis was reperformed to decrease the heterogeneity of causal effects (18). Finally, a leave-one-out analysis was conducted as a sensitivity analysis. The leave-one-out method assesses the independent impact of each SNP on the outcome (19). This approach aimed to obtain more robust results.

## Results

In exploring the genetically predicted causal association of digestive diseases as exposure factors on the risk of cerebrovascular disease, a series of IVs’ selecting processes were performed and a total of 376 SNPs were extracted from the GWAS summary data of 12 digestive system disorders, ranging from 1 to 121 SNPs. In exploring the genetically predicted causal association of metabolic factors on the risk of cerebrovascular disease, a total of 382 SNPs were extracted from the GWAS summary data of SBP, BMI and T2D, respectively. All *F*-statistic >10. The detailed information of IVs extracted for all exposure factors is shown in Supplementary Table S1.

### Results of TSMR

#### MR results of digestive system diseases on stroke

**Figure 2:**
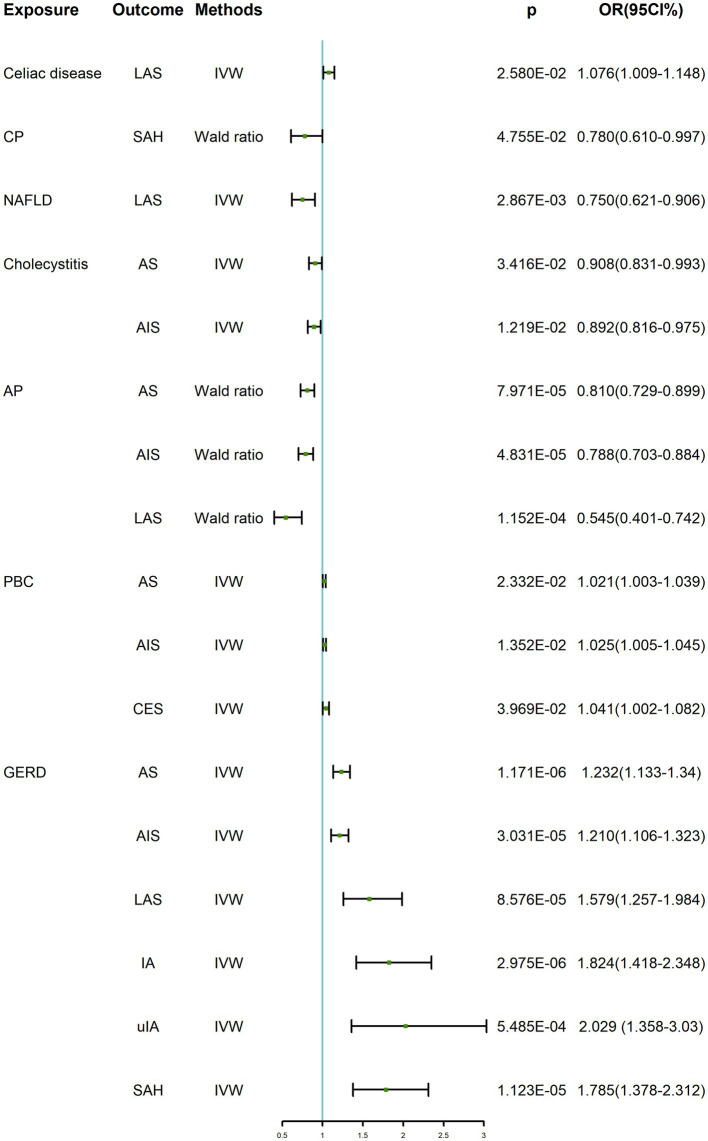
TSMR positive results of digestive system diseases on cerebrovascular diseases. GERD, gastroesophageal reflux disease; PBC, primary biliary cholangitis; AP, acute pancreatitis; CP, chronic pancreatitis; AS, any stroke; AIS, any ischemic stroke; CES, cardioembolic stroke; LAS, large-artery atherosclerotic stroke; IA, intracranial aneurysm; uIA, unruptured intracranial aneurysm; SAH, subarachnoid hemorrhage; IVW, inverse variance weighted; OR, odds ratio; CI, confidence interval.

As shown in Table 2; Supplementary Tables S2, S3; Figure 2, when genetically predicted digestive system diseases were considered as the exposure factor, we observed significant positive causal relationships between gastroesophageal reflux disease (GERD) and AS (OR: 1.232, 95% CI: 1.133–1.340, *p* = 1.171 × 10^−6^), AIS (OR: 1.210, 95% CI: 1.106–1.323, *p* = 3.031 × 10^−5^), and LAS (OR: 1.579, 95% CI: 1.257–1.984, *p* = 8.576 × 10^−5^), with no evidence of heterogeneity or horizontal pleiotropy. Additionally, genetically predicted primary biliary cholangitis showed suggestive causal relationships with AS (OR: 1.021, 95% CI: 1.003–1.039, *p* = 0.023), AIS (OR: 1.025, 95% CI: 1.005–1.045, *p* = 0.014), and CES (OR: 1.041, 95% CI: 1.002–1.082, *p* = 0.040), with no observed heterogeneity or horizontal pleiotropy. And, cholecystitis demonstrated suggestive causal relationships with AS (OR: 0.908, 95% CI: 0.831–0.993, *p* = 0.034) and AIS (OR: 0.892, 95% CI: 0.816–0.975, *p* = 0.012), without heterogeneity, but the test for horizontal pleiotropy could not be performed with only including 2 SNPs. In the IVW method, a suggestive positive causal relationship between celiac disease and LAS (OR: 1.076, 95% CI: 1.009–1.148, *p* = 0.026) was observed, without horizontal pleiotropy but with heterogeneity so the weighted median method results were considered as evaluation method. However, this suggestive causal relationship disappeared in the weighted median method, indicating no causal relationship between genetic prediction of celiac disease and LAS. Genetically predicted NAFLD showed a suggestive negative causal relationship with LAS (OR: 0.750, 95% CI: 0.621–0.906, *p* = 0.003), with no observed heterogeneity. However, due to only including 2 available SNPs, the test of horizontal pleiotropy was not performed. Genetically predicted acute pancreatitis exhibited significant negative causal relationships with AS (OR: 0.810, 95% CI: 0.729–0.899, *p* = 7.971 × 10^−5^), AIS (OR: 0.788, 95% CI: 0.703–0.884, *p* = 4.831 × 10^−5^), and LAS (OR: 0.545, 95% CI: 0.401–0.742, *p* = 1.152 × 10^−4^). However, considering only one IV, the MR-Egger intercept and Cochran’s *Q* tests for its pleiotropy and heterogeneity were not feasible, and visualization of the results was not possible. Therefore, no definitive causal relationship between acute pancreatitis and AS, AIS, and LAS can be concluded.

**Table 2:** TSMR positive results of digestive system diseases on cerebrovascular diseases.

Exposures	Outcome	Methods	SNP	*p*val	OR (95% CI)	Cochran’s IVW_*p*	MR-Egger intercept_*p*
AP	AS	Wald ratio	1	7.971 ×10^−5^	0.810 (0.729–0.899)	—	—
Cholecystitis	AS	IVW	2	3.416 ×10^−2^	0.908 (0.831–0.993)	0.100	—
GERD	AS	IVW	66	1.171 ×10^−6^	1.232 (1.133–1.34)	0.068	0.641
PBC	AS	IVW	33	2.332 ×10^−2^	1.021 (1.003–1.039)	0.126	0.213
AP	AIS	Wald ratio	1	4.831 ×10^−5^	0.788 (0.703–0.884)	—	—
Cholecystitis	AIS	IVW	2	1.219 ×10^−2^	0.892 (0.816–0.975)	0.134	—
GERD	AIS	IVW	66	3.031 ×10^−5^	1.210 (1.106–1.323)	0.116	0.822
PBC	AIS	IVW	33	1.352 ×10^−2^	1.025 (1.005–1.045)	0.103	0.275
PBC	CES	IVW	33	3.969 ×10^−2^	1.041 (1.002–1.082)	0.073	0.354
AP	LAS	Wald ratio	1	1.152 ×10^−4^	0.545 (0.401–0.742)	—	—
Celiac disease	LAS	IVW	34	2.580 ×10^−2^	1.076 (1.009–1.148)	0.001	0.859
GERD	LAS	IVW	66	8.576 ×10^−5^	1.579 (1.257–1.984)	0.062	0.881
NAFLD	LAS	IVW	2	2.867 ×10^−3^	0.750 (0.621–0.906)	0.526	—
GERD	IA	IVW	56	2.975 ×10^−6^	1.824 (1.418–2.348)	0.001	0.424
GERD	uIA	IVW	56	5.485 ×10^−4^	2.029 (1.358–3.03)	0.038	0.380
CP	SAH	Wald ratio	1	4.755 ×10^−2^	0.780 (0.610–0.997)	—	—
GERD	SAH	IVW	56	1.123 ×10^−5^	1.785 (1.378–2.312)	0.087	0.086

GERD, gastroesophageal reflux disease; PBC, primary biliary cholangitis; AP, acute pancreatitis; CP, chronic pancreatitis; AS, any stroke; AIS, any ischemic stroke; CES, cardioembolic stroke; LAS, large-artery atherosclerotic stroke; IA, intracranial aneurysm; uIA, unruptured intracranial aneurysm; SAH, subarachnoid hemorrhage; IVW, inverse variance weighted; OR, odds ratio; CI, confidence interval.

#### MR results of digestive system diseases on intracranial aneurysms

In the IVW, we found that genetically predicted GERD presented significant positive causality with IA (OR: 1.824, 95% CI: 1.418–2.348, *p* = 2.975 × 10^−6^) and SAH (OR: 1.785, 95% CI: 1.378–2.312, *p* = 1.123 × 10^−5^), and suggestive positive causality with uIA (OR: 2.029, 95% CI: 1.358–3.030, *p* = 5.485 × 10^−4^). However, heterogeneity was found for both IA and uIA analyses, so the weighted median was suggestive; this causality remained under the weighted median approach, so the results were considered robust. In all analyses, there was no horizontal pleiotropy. In addition, there was a suggestive causal relationship between genetically predicted chronic pancreatitis and SAH (OR: 0.780, 95% CI: 0.610–0.997, *p* = 0.048), which was not considered to be established. Similarly, there was only one SNP, heterogeneity and horizontal pleiotropy could not be detected, and visualization of the results was not possible. All results were shown in Table 2; Supplementary Tables S4, S5; Figure 2.

### Results of multivariate MR analysis

**Figure 3:**
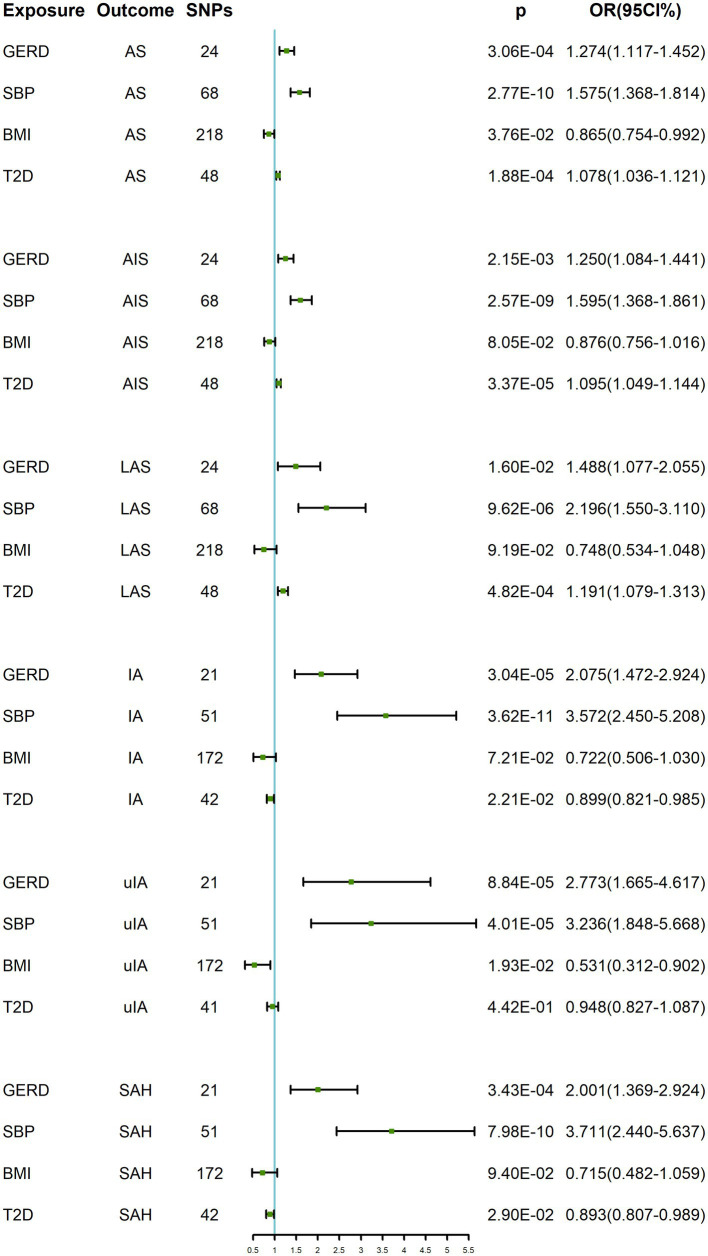
MVMR results of GERD on cerebrovascular diseases after adjusting for BMI, SBP and T2D. GERD, gastroesophageal reflux disease; SBP, systolic blood pressure; BMI, body mass index; T2D, type 2 diabetes; AS, any stroke; AIS, any ischemic stroke; LAS, large-artery atherosclerotic stroke; IA, intracranial aneurysm; uIA, unruptured intracranial aneurysm; SAH, subarachnoid hemorrhage; OR, odds ratio; CI, confidence interval.

As shown in Table 3 and Figure 3, in the multivariate MR analysis, it was found that when adjusted for BMI, SBP and T2D, GERD was still associated with AS (OR: 1.274, 95% CI: 1.117–1.452, *p* = 3.06 × 10^−4^), AIS (OR: 1.250, 95% CI: 1.084–1.441, *p* = 2.15 × 10^−3^), LAS (OR: 1.488, 95% CI: 1.077–2.055, *p* = 1.60 × 10^−2^), IA (OR: 2.075, 95% CI: 1.472–2.924, *p* = 3.04 × 10^−5^), uIA (OR: 2.773, 95% CI: 1.665–4.617, *p* = 8.84 × 10^−5^), and SAH (OR: 1.784, 95% CI: 1.231–2.585, *p* = 3.43 × 10^−4^) showed a positive causal relationship.

**Table 3:** MVMR results of GERD on cerebrovascular diseases after adjusting for BMI, SBP and T2D.

Exposures	Outcomes	SNP	*p*val	OR (95% CI)
GERD	AS	24	3.06 × 10^−4^	1.274 (1.117–1.452)
SBP	AS	68	2.77 × 10^−10^	1.575 (1.368–1.814)
BMI	AS	218	3.76 × 10^−2^	0.865 (0.754–0.992)
T2D	AS	48	1.88 × 10^−4^	1.078 (1.036–1.121)
GERD	AIS	24	2.15 × 10^−3^	1.250 (1.084–1.441)
SBP	AIS	68	2.57 × 10^−9^	1.595 (1.368–1.861)
BMI	AIS	218	8.05 × 10^−2^	0.876 (0.756–1.016)
T2D	AIS	48	3.37 × 10^−5^	1.095 (1.049–1.144)
GERD	LAS	24	1.60 × 10^−2^	1.488 (1.077–2.055)
SBP	LAS	68	9.62 × 10^−6^	2.196 (1.550–3.110)
BMI	LAS	218	9.19 × 10^−2^	0.748 (0.534–1.048)
T2D	LAS	48	4.82 × 10^−4^	1.191 (1.079–1.313)
GERD	IA	21	3.04 × 10^−5^	2.075 (1.472–2.924)
SBP	IA	51	3.62 × 10^−11^	3.572 (2.450–5.208)
BMI	IA	172	7.21 × 10^−2^	0.722 (0.506–1.030)
T2D	IA	42	2.21 × 10^−2^	0.899 (0.821–0.985)
GERD	uIA	21	8.84 × 10^−5^	2.773 (1.665–4.617)
SBP	uIA	51	4.01 × 10^−5^	3.236 (1.848–5.668)
BMI	uIA	172	1.93 × 10^−2^	0.531 (0.312–0.902)
T2D	uIA	41	4.42 × 10^−1^	0.948 (0.827–1.087)
GERD	SAH	21	3.43 × 10^−4^	2.001 (1.369–2.924)
SBP	SAH	51	7.98 × 10^−10^	3.711 (2.440–5.637)
BMI	SAH	172	9.40 × 10^−2^	0.715 (0.482–1.059)
T2D	SAH	42	2.90 × 10^−2^	0.893 (0.807–0.989)

GERD, gastroesophageal reflux disease; SBP, systolic blood pressure; BMI, body mass index; T2D, type 2 diabetes; AS, any stroke; AIS, any ischemic stroke; LAS, large-artery atherosclerotic stroke; IA, intracranial aneurysm; uIA, unruptured intracranial aneurysm; SAH, subarachnoid hemorrhage; OR, odds ratio; CI, confidence interval.

### Results of two-step MR analysis

#### MR results of GERD on metabolic factors

When genetically predicted GERD was used as an exposure factor, a positive causal relationship was found with both SBP (OR: 1.088, 95% CI: 1.057–1.120, *p* = 1.27 × 10^−8^), BMI (OR: 1.242, 95% CI: 1.200–1.286, *p* = 2.44 × 10^−34^) and T2D (OR: 1.688, 95% CI: 1.469–1.938, *p* = 1.40 × 10^−13^), although there was heterogeneity, the results of the weighted median approach remained significant and no horizontal pleiotropy was detected (Table 4), so the results were considered robust.

**Table 4:** TSMR results of GERD on metabolic factors.

Exposures	Outcome	Methods	*p*val	OR (95% CI)	Heterogeneity test	Pleiotropy test
					Cochran’s IVW_*p*	MR-Egger intercept_*p*
GERD	SBP	MR Egger	6.45 × 10^−2^	1.188 (0.993–1.421)	3.20 × 10^−14^	0.334
		WM	7.74 × 10^−6^	1.068 (1.038–1.100)		
		IVW	1.27 × 10^−8^	1.088 (1.057–1.120)		
GERD	BMI	MR Egger	3.42 × 10^−1^	1.136 (0.876–1.473)	3.91 × 10^−6^	0.501
		WM	3.83 × 10^−18^	1.196 (1.149–1.246)		
		IVW	2.44 × 10^−34^	1.242 (1.200–1.286)		
GERD	T2D	MR Egger	3.29 × 10^−2^	2.550 (1.098–5.924)	2.48 × 10^−42^	0.333
		WM	7.21 × 10^−21^	1.649 (1.485–1.831)		
		IVW	1.40 × 10^−13^	1.688 (1.469–1.938)		

GERD, gastroesophageal reflux disease; SBP, systolic blood pressure; BMI, body mass index; T2D, type 2 diabetes; OR, odds ratio; CI, confidence interval; IVW, inverse variance weighted; WM, weighted median.

#### MR results of metabolic factors on cerebrovascular disease

When stroke was considered as the outcome, it was found that genetically predicted SBP had a positive causal relationship with AS (OR: 1.740, 95% CI: 1.562–1.938, *p* = 8.743 × 10^−24^), AIS (OR: 1.766, 95% CI: 1.575–1.981, *p* = 2.649 × 10^−22^), and LAS (OR: 2.826, 95% CI: 2.212–3.612, *p* = 1.009 × 10^−16^). When genetically predicted BMI was considered as the exposure factor, a positive causal relationship was observed with AS (OR: 1.206, 95% CI: 1.128–1.290, *p* = 5.184 × 10^−8^), AIS (OR: 1.216, 95% CI: 1.130–1.309, *p* = 1.887 × 10^−7^), and LAS (OR: 1.429, 95% CI: 1.191–1.715, *p* = 1.245 × 10^−4^). When genetically predicted T2D was considered as the exposure factor, a positive causal relationship was observed with AS (OR: 1.086, 95% CI: 1.054–1.119, *p* = 5.69 × 10^−8^), AIS (OR: 1.091, 95% CI: 1.058–1.126, *p* = 5.37 × 10^−8^), and LAS (OR: 1.219, 95% CI: 1.118–1.330, *p* = 7.36 × 10^−6^). Although there was evidence of heterogeneity in the above analyses, the results of the weighted median method remained significant, and no horizontal pleiotropy was detected (Table 5). Therefore, the results were considered robust.

**Table 5:** TSMR results of metabolic factors on cerebrovascular diseases.

Exposures	Outcomes	Methods	*p*val	OR (95% CI)	Heterogeneity test	Pleiotropy test
					IVW_*p*	MR-Egger intercept_*p*
SBP	AS	MR Egger	1.19 × 10^−4^	1.921 (1.386–2.663)	8.44 × 10^−12^	0.528
		WM	3.63 × 10^−17^	1.765 (1.546–2.014)		
		IVW	8.74 × 10^−24^	1.740 (1.562–1.938)		
SBP	AIS	MR Egger	1.35 × 10^−4^	1.986 (1.405–2.806)	2.81 × 10^−10^	0.482
		WM	1.51 × 10^−15^	1.793 (1.553–2.069)		
		IVW	2.65 × 10^−22^	1.766 (1.575–1.981)		
SBP	LAS	MR Egger	4.17 × 10^−4^	3.891 (1.851–8.178)	2.66 × 10^−3^	0.372
		WM	1.28 × 10^−6^	2.331 (1.655–3.283)		
		IVW	1.01 × 10^−16^	2.826 (2.212–3.612)		
SBP	IA	MR Egger	1.28 × 10^−4^	5.911 (2.437–14.334)	8.24 × 10^−8^	0.189
		WM	4.59 × 10^−12^	3.640 (2.524–5.248)		
		IVW	9.61 × 10^−17^	3.361 (2.525–4.474)		
SBP	uIA	MR Egger	4.52 × 10^−3^	6.180 (1.791–21.323)	1.43 × 10^−1^	0.276
		WM	3.57 × 10^−6^	3.910 (2.197–6.960)		
		IVW	2.06 × 10^−8^	3.220 (2.139–4.845)		
SBP	SAH	MR Egger	1.75 × 10^−5^	8.858 (3.379–23.226)	1.82 × 10^−6^	0.079
		WM	1.96 × 10^−9^	3.438 (2.297–5.146)		
		IVW	1.35 × 10^−16^	3.901 (2.825–5.386)		
BMI	AS	MR Egger	1.01 × 10^−1^	1.163 (0.972–1.393)	6.23 × 10^−5^	0.670
		WM	1.02 × 10^−2^	1.151 (1.034–1.281)		
		IVW	5.18 × 10^−8^	1.206 (1.128–1.290)		
BMI	AIS	MR Egger	4.13 × 10^−2^	1.228 (1.009–1.494)	5.76 × 10^−5^	0.916
		WM	1.99 × 10^−2^	1.156 (1.023–1.307)		
		IVW	1.89 × 10^−7^	1.216 (1.130–1.309)		
BMI	LAS	MR Egger	6.12 × 10^−2^	1.595 (0.980–2.597)	4.83 × 10^−5^	0.633
		WM	1.26 × 10^−3^	1.615 (1.207–2.162)		
		IVW	1.25 × 10^−4^	1.429 (1.191–1.715)		
BMI	IA	MR Egger	5.58 × 10^−1^	1.139 (0.738–1.757)	3.13 × 10^−2^	0.400
		WM	4.31 × 10^−2^	1.336 (1.009–1.77)		
		IVW	2.78 × 10^−4^	1.353 (1.150–1.593)		
BMI	uIA	MR Egger	3.04 × 10^−1^	0.686 (0.335–1.406)	6.58 × 10^−1^	0.067
		WM	3.73 × 10^−1^	1.256 (0.761–2.073)		
		IVW	7.25 × 10^−2^	1.280 (0.978–1.677)		
BMI	SAH	MR Egger	5.78 × 10^−1^	1.159 (0.690–1.947)	7.44 × 10^−3^	0.662
		WM	1.54 × 10^−1^	1.277 (0.913–1.788)		
		IVW	1.06 × 10^−2^	1.290 (1.061–1.568)		
T2D	AS	MR Egger	1.00 × 10^−2^	1.097 (1.024–1.176)	5.48 × 10^−2^	0.750
		WM	5.91 × 10^−4^	1.092 (1.038–1.148)		
		IVW	5.69 × 10^−8^	1.086 (1.054–1.119)		
T2D	AIS	MR Egger	5.57 × 10^−3^	1.113 (1.033–1.198)	1.24 × 10^−1^	0.575
		WM	2.34 × 10^−4^	1.107 (1.049–1.169)		
		IVW	5.37 × 10^−8^	1.091 (1.058–1.126)		
T2D	LAS	MR Egger	2.90 × 10^−3^	1.368 (1.118–1.673)	2.61 × 10^−3^	0.219
		WM	1.99 × 10^−4^	1.255 (1.114–1.415)		
		IVW	7.36 × 10^−6^	1.219 (1.118–1.330)		
T2D	IA	MR Egger	2.36 × 10^−2^	0.803 (0.666–0.968)	1.44 × 10^−6^	0.006
		WM	3.71 × 10^−1^	0.945 (0.835–1.070)		
		IVW	6.47 × 10^−1^	1.021 (0.935–1.114)		
T2D	uIA	MR Egger	1.78 × 10^−1^	0.836 (0.645–1.083)	3.94 × 10^−1^	0.100
		WM	9.58 × 10^−1^	1.006 (0.807–1.254)		
		IVW	7.80 × 10^−1^	1.017 (0.904–1.144)		
T2D	SAH	MR Egger	3.92 × 10^−2^	0.804 (0.655–0.986)	1.95 × 10^−4^	0.009
		WM	2.53 × 10^−1^	0.920 (0.798–1.061)		
		IVW	5.46 × 10^−1^	1.030 (0.936–1.134)		

SBP, systolic blood pressure; BMI, body mass index; T2D, type 2 diabetes; AS, any stroke; AIS, any ischemic stroke; LAS, large-artery atherosclerotic stroke; IA, intracranial aneurysm; uIA, unruptured intracranial aneurysm; SAH, subarachnoid hemorrhage; OR, odds ratio; CI, confidence interval; IVW, inverse variance weighted; WM, weighted median.

When intracranial aneurysm was considered as the outcome, it was found that SBP had a positive causal relationship with IA (OR: 3.361, 95% CI: 2.525–4.474, *p* = 9.605 × 10^−17^), unruptured IA (uIA) (OR: 3.220, 95% CI: 2.139–4.845, *p* = 2.055 × 10^−8^), and subarachnoid hemorrhage (SAH) (OR: 3.901, 95% CI: 2.825–5.386, *p* = 1.353 × 10^−16^). In the MR analysis with IA and SAH, there was heterogeneity but no horizontal pleiotropy. The results of the weighted median method still showed a significant causal relationship between them, indicating the robustness of the results. Genetically predicted BMI had a positive causal relationship with IA (OR: 1.353, 95% CI: 1.150–1.593, *p* = 2.779 × 10^−4^), with heterogeneity, but the MR results of the weighted median method remained significant, and no horizontal pleiotropy was found. Therefore, the results were considered robust. Although genetically predicted BMI had a significant causal relationship with SAH (OR: 1.290, 95% CI: 1.061–1.568, *p* = 0.011), there was heterogeneity. When evaluated using the weighted median method, the significance disappeared, and therefore, a causal effect between BMI and SAH could not be concluded. There was no causal relationship between genetically predicted BMI and uIA (OR: 1.280, 95% CI: 0.978–1.677, *p* = 0.07), and no heterogeneity or horizontal pleiotropy was observed. However, the significant causal relationship were not found in T2D on IA, uIA, and SAH (Table 5).

### Results mediation MR analysis

Through two-step MR analysis, it was found that when the outcome was stroke, the causal relationship between GERD and AS, AIS, and LAS was partially mediated by SBP and BMI. Specifically, the mediated effects of GERD, mediated by SBP, on AS, AIS, and LAS were 0.047, 0.048, and 0.087, accounting for 22.49, 25.13, and 19.04% of the total effects, respectively. The mediated effects of GERD, mediated by BMI, on AS, AIS, and LAS were 0.041, 0.043, and 0.077, accounting for 19.62, 22.51, and 16.85% of the total effects, respectively. The mediated effects of GERD, mediated by T2D, on AS, AIS, and LAS were 0.043, 0.046, and 0.104, accounting for 20.57, 24.08, and 22.76% of the total effects, respectively (Table 6).

**Table 6:** The results of mediation MR analysis.

Exposure	Mediation	Outcome	*β*1	*β*2	*β*3	Mediating effect	Mediating ratio
GERD	SBP	AS	0.209	0.084	0.554	0.047	22.49%
		AIS	0.191		0.569	0.048	25.13%
		LAS	0.457		1.039	0.087	19.04%
		IA	0.601		1.212	0.102	16.97%
		uIA	0.707		1.169	0.098	13.86%
		SAH	0.58		1.361	0.114	19.66%
	BMI	AS	0.209	0.217	0.188	0.041	19.62%
		AIS	0.191		0.195	0.043	22.51%
		LAS	0.457		0.357	0.077	16.85%
		IA	0.601		0.303	0.066	10.98%
		SAH	0.58		0.255	0.055	9.48%
	T2D	AS	0.209	0.523	0.083	0.043	20.57%
		AIS	0.191		0.087	0.046	24.08%
		LAS	0.457		0.198	0.104	22.76%

SBP, systolic blood pressure; BMI, body mass index; T2D, type 2 diabetes; AS, any stroke; AIS, any ischemic stroke; LAS, large-artery atherosclerotic stroke; IA, intracranial aneurysm; uIA, unruptured intracranial aneurysm; SAH, subarachnoid hemorrhage.

When the outcome was intracranial aneurysm, SBP and BMI were also identified as factors mediating the causal relationship between GERD and intracranial aneurysm. Specifically, the mediated effects of GERD, mediated by SBP, on IA, unruptured IA (uIA), and subarachnoid hemorrhage (SAH) were 0.102, 0.098, and 0.114, accounting for 16.97, 13.86, and 19.66% of the total effects, respectively. The mediated effects of GERD, mediated by BMI, on IA and SAH were 0.066 and 0.055, accounting for 10.98 and 9.48% of the total effects, respectively (Table 6).

## Discussion

This MR study investigated the causal link between digestive system diseases and the development of cerebrovascular diseases. The research findings revealed a significant causal association between gastroesophageal reflux disease (GERD) and the risk of cerebrovascular diseases. This insight underscores the genetic predisposition of GERD as a major risk factor for cerebrovascular conditions. The study’s sensitivity analysis demonstrated consistent and robust causal effects. Moreover, in the multivariable MR analysis, even upon adjusting for variables like BMI, systolic blood pressure (SBP), and type 2 diabetes (T2D), the causal relationship between GERD and cerebrovascular diseases remained evident. Additionally, the two-step MR analysis outcomes indicated that BMI, SBP, and T2D potentially act as partial mediators in the causal pathway linking GERD to cerebrovascular diseases. Therefore, this paragraph will focus solely on discussing the relationship between GERD and cerebrovascular disease.

Our MR findings are consistent with previous studies on the causal relationship between GERD and cerebrovascular diseases. Sheu et al. (20) explored the risk of stroke in young individuals within a year of GERD diagnosis. They reported that after accounting for other stroke risk factors, young individuals with GERD had a 1.68 times higher likelihood of experiencing a stroke during the one-year follow-up period compared to age- and gender-matched controls. Additionally, Jansson et al. (21) proposed a plausible link between stroke and GERD based on a population-based cross-sectional study conducted in Norway.

Several mechanisms have been proposed to explain the potential links between GERD and the increased risk of cerebrovascular diseases. Firstly, *Helicobacter pylori* (*H. pylori*) infection is a crucial mechanism. *H. pylori*, known as a high-risk factor for digestive diseases (22), can lead to conditions such as peptic ulcer and gastric cancer. The impact of *H. pylori* on the development of GERD may involve alterations in gastroesophageal barrier function, changes like reflux, or effects on gastric emptying (23). A study involving 156 patients with peptic ulcer and reflux esophagitis demonstrated that the eradication of *H. pylori* significantly improved GERD symptoms when compared to patients with persistent infection (24). Furthermore, cross-sectional studies have suggested a potential association between *H. pylori* infection and conditions like atherosclerotic coronary artery disease (25, 26) or ischemic stroke (27, 28). *H. pylori* can persistently infect and survive in the gastric mucosa, triggering acute and chronic inflammatory responses (22, 29, 30). These inflammatory responses can subsequently initiate and promote atherosclerosis (31). *H. pylori* can be categorized into two main strains depending on the presence or absence of the cytotoxin-associated gene-A (CagA) gene. Type 1 *H. pylori*, which possesses the CagA gene, expresses both CagA and vacuolating cytotoxin-A (VacA), whereas type 2 *H. pylori* lacks its expression (32). VacA has been identified to bind to cell membrane receptors, be internalized by cells, translocate to mitochondria, and trigger inflammation and apoptosis (33). Furthermore, GroEL, a newly recognized virulence factor in *H. pylori*, acts as a molecular chaperone protein essential for the correct folding of proteins in the bacterium (34). Studies have shown that GroEL also plays a role in the gastric colonization of *H. pylori* and subsequent initiation of the inflammatory response (35). A MR study examining the causal relationship between *H. pylori* and stroke revealed that the link between *H. pylori* and stroke was solely mediated by the pro-inflammatory factor C-reactive protein (CRP) (36). Previous research has consistently demonstrated that elevated CRP levels are correlated with an elevated risk of cardiovascular disease and stroke (37, 38). Several studies have demonstrated that elevated neutrophil counts are linked to increased severity of infarcts and poorer neurological outcomes (39–42). Moreover, *H. pylori* has the ability to recruit various immune cells, including neutrophils, macrophages, dendritic cells, T and B cells, and IL-8 (43–46). Several mechanisms may contribute to the association between leukocyte counts and increased infarct severity. Atherosclerosis is increasingly viewed as a chronic inflammatory disease (47), and one possibility is that elevated leukocyte counts precede stroke episodes, reflecting the burden of atherosclerotic disease. Leukocytosis is associated with the degree of atherosclerosis (48, 49), is a risk factor for cardiovascular events and stroke (50, 51), and is associated with stroke subtypes (50). In addition, there is evidence that leukocytosis may be associated with plaque instability and induction of acute thrombotic events. Secondly, GERD-induced stimulation of the esophagus and stomach can lead to impaired cardiac conduction signals or autonomic regulation, potentially causing arrhythmias (52). Additionally, GERD-related reflexes, such as the esophagus-heart reflex, could contribute to coronary vasoconstriction and reduced coronary blood flow, potentially leading to cardioembolic stroke (53). Thirdly, In GERD, there is a high prevalence of vagal nerve dysfunction, which is associated with delayed esophageal transit, abnormal peristalsis, and an increased frequency of transient lower esophageal sphincter relaxation (54, 55). Because the immune system directly controls the vagal nerve through the cholinergic anti-inflammatory pathway, dysfunction of the vagal nerve may trigger an exaggerated inflammatory response and the spread of inflammatory mediators into the bloodstream, potentially initiating the common atherosclerotic process and leading to cardiovascular events (56). Finally, cardiovascular autonomic dysfunction mediated by the vagus nerve in GERD patients may lead to an imbalance in the sympathetic and parasympathetic nervous systems in the cerebrovascular system. This alteration could result in a defect in the autoregulation of cerebral blood flow, thereby increasing the risk of cerebrovascular diseases (57, 58). As GERD can seriously increase the risk of atherosclerosis, it can seriously increase the burden on blood vessels, resulting in an increased prevalence of aneurysms.

Several pathological cascades of obesity-induced responses can lead to thrombosis and cardiovascular disease. Obesity is associated with increased inflammatory markers, leading to a state of low-grade chronic inflammation (59). This low-grade inflammation and systemic oxidative stress can be deleterious to endothelial cells, pushing them toward a prothrombotic state. Mechanisms such as platelet reactivity, enhanced coagulation, and impaired fibrinolysis are recognized contributors to this process (60). The most intuitive pathways involve elevated blood pressure and diabetes mellitus, both established causative factors for atherosclerosis in both large and small vessels (61). Furthermore, studies have shown that GERD is associated with various risk factors for vascular disease, including BMI, T2D, SBP, smoking, triglycerides (TG), and insomnia. GERD and obesity often coexist, with weight loss potentially improving GERD symptoms (62). Meta-analysis studies conducted in the US have identified a positive association between increased BMI and GERD incidence (63). Additionally, a recent Mendelian randomization study linked genetically predicted higher BMI, T2D, and smoking to an elevated risk of GERD (64). Turning to diabetes, it is a recognized risk factor for stroke and vascular disease through multiple mechanisms. These include increased production of free oxygen radicals and oxidative stress (65), heightened glycosylation product production (66), increased aldose reductase activity in the polyol pathway (65, 67), and activation of specific protein kinase C (PKC) isoforms (67). Diabetes can also contribute to cerebrovascular disease by inducing autonomic neuropathy, which in turn impacts cerebrovascular sympathetic and parasympathetic innervation (68, 69). Autonomic neuropathy leads to inadequate blood flow autoregulation, rendering cerebral vessels more vulnerable to damage and occlusion (70, 71). Evidence has demonstrated that diabetic autonomic neuropathy causes a consistent rise in sympathetic tone at night, resulting in elevated heart rate and blood pressure levels (72). Additionally, hypertension, a prevalent cardiovascular risk factor, is provoked by prolonged high blood pressure, promoting atherosclerosis and vascular remodeling that leads to thickened arterial walls (73). Hypertension also induces cardiac morphological changes such as left ventricular hypertrophy and left atrial dilatation, which are independent risk factors for stroke (74–76).

In addition, recent research has intensified the focus on the microbial-gut-brain axis, particularly in exploring the interplay between gut microbiota and GERD. Liu et al. (77) observed a lower prevalence of the Actinobacteria phylum among individuals with GERD (78), consistent with the findings of Wang’s et al. (79) MR analysis. Specifically, in a study involving pediatric patients with GERD, higher levels of *Aspergillus* and *Anaplasma* phyla were detected, while concentrations of Thickettsia and Actinobacteria phyla were notably reduced (80). Notably, a Japanese study employed a novel method utilizing quantitative 16S rRNA gene PCR to assess total bacterial counts. The results indicated that the relative proportions of taxa such as *Aspergillus*, Thick-walled Bacteria, *Anaplasma*, *Clostridium*, and Actinobacteria exhibited stronger correlations with esophageal diseases than absolute bacterial counts (77). Moreover, a separate MR study revealed a causal link between gut microbes and cerebrovascular disease (19), paving the way for further investigation into the mechanisms of the microbe-gut-brain axis.

In this study, we utilized published GWAS summary data to conduct a thorough MR analysis. This approach enabled us to assess the causal association between digestive system diseases and cerebrovascular diseases more effectively, eliminating confounding factors and reverse causality commonly found in traditional observational studies. Our study benefitted from the substantial sample sizes of all GWAS data and the genetic homogeneity of European populations included, thereby mitigating concerns related to population stratification and genetic variation. Furthermore, we employed sensitivity analysis to enhance the reliability and robustness of our MR findings. Nevertheless, it is important to acknowledge the presence of certain limitations in our study. Firstly, using instruments with an F-statistic >10 only reduces bias to less than a certain level, and the issues with weak instrument bias still occur (81). Secondly, there was significant heterogeneity present in some of the analyses which relate to directional pleiotropy. Confounding by cryptic pleiotropy is a known limitation of MR analyses (82). However, we addressed this possibility by quantifying pleiotropy and by using multiple MR approaches with different modeling assumptions regarding the use of pleiotropic variants in the analyses to further strengthen the validity of our MR models. Finally, there are other limitations, such as the generalizability of the conclusions to other ethnicities and the inability to assess selection bias due to the lack of individual-level data for binary variables.

## Conclusion

This study indicates a clear positive causal relationship between GERD and cerebrovascular diseases. Moreover, this causal association remains significant even after adjusting for BMI, SBP and T2D. The mediation MR analysis suggests that BMI, SBP and T2D may mediate the causal relationship between GERD and the risk of cerebrovascular diseases.

## Data availability statement

The original contributions presented in the study are included in the article/Supplementary material, further inquiries can be directed to the corresponding author.

## Ethics statement

Ethical review and approval was not required for the study on human participants in accordance with the local legislation and institutional requirements. Written informed consent from the patients/participants or patients/participants’ legal guardian/next of kin was not required to participate in this study in accordance with the national legislation and the institutional requirements.

## Author contributions

HQ: Conceptualization, Data curation, Formal analysis, Methodology, Resources, Software, Supervision, Validation, Visualization, Writing – original draft, Writing – review & editing. SS: Supervision, Validation, Writing – review & editing. FY: Supervision, Validation, Writing – review & editing. PH: Data curation, Methodology, Resources, Writing – review & editing. XZ: Supervision, Writing – review & editing.

## Footnotes

^1^http://www.phenoscanner.medschl.cam.ac.uk/
